# First person – Kevin Aguirre-Carvajal

**DOI:** 10.1242/bio.062564

**Published:** 2026-03-25

## Abstract

First Person is a series of interviews with the first authors of a selection of papers published in Biology Open, helping researchers promote themselves alongside their papers. Kevin Aguirre-Carvajal is first author on ‘ What impact do new homologs have on detecting interdomain horizontal gene transfer in eukaryotes? A reassessment of Katz (2015)’, published in BiO. Kevin is a PhD student in the lab of Dr Vinicio Armijos-Jaramillo at Universidad de Las Américas, Quito, Ecuador, and is pursuing his PhD in collaboration with the Universidade da Coruña, Coruña, Spain, using computational and machine learning approaches to extract biological insights from large-scale genomic data.

**Figure:**
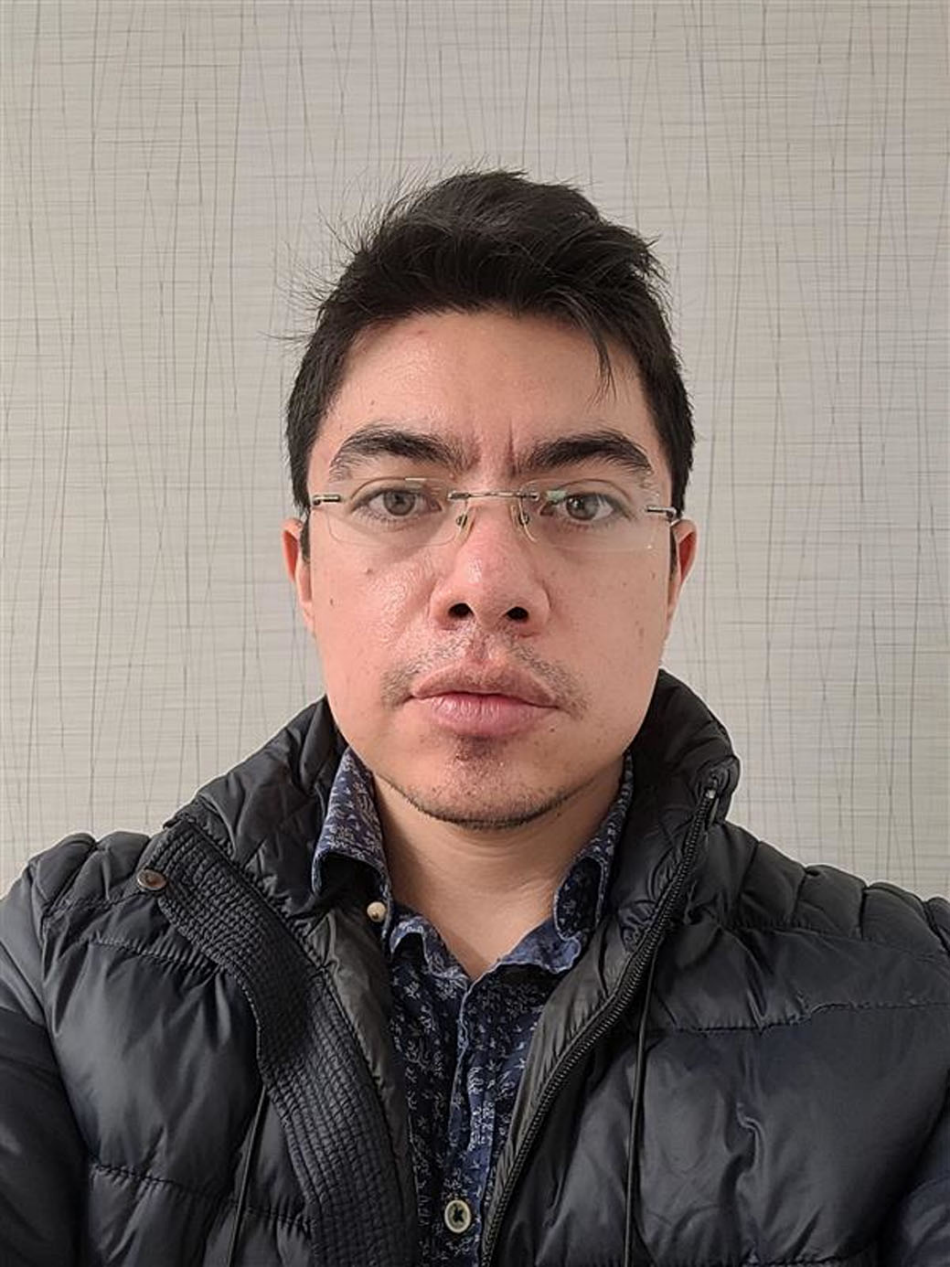
Kevin Aguirre-Carvajal

**Describe your scientific journey and your current research focus**

My scientific journey has been somewhat unconventional. I studied Biotechnology Engineering in Ecuador; but, during my undergraduate thesis, I realized that I did not particularly enjoy laboratory work. After graduating during the COVID-19 pandemic, I struggled to find job opportunities in my field, which are quite limited in Ecuador. At that time, I even considered changing career paths and pursuing a Master's degree in a completely different area. Before making that decision, I decided to give science one last chance. I contacted several researchers and eventually had the opportunity to meet Dr Vinicio Armijos-Jaramillo at the Universidad de Las Américas. Although I had little experience in bioinformatics at the time, he gave me the opportunity to join his group. I started working with him as a volunteer and quickly discovered that I truly enjoyed computational biology. I put a great deal of effort into learning the field, and over time this collaboration evolved into a formal research position. In parallel, I completed a Master's degree in Bioinformatics at the Universidad Europea de Madrid. Currently, I am pursuing a PhD at the Universidade da Coruña, where my research focuses on applying bioinformatics and machine learning approaches to study genome evolution and detect signals of horizontal gene transfer.

**Who or what inspired you to become a scientist?**

I have always been driven by curiosity. The idea of discovering new things and understanding how nature works strongly attracted me to science. I also enjoy sharing knowledge with others, and science provides a way to both explore new ideas and communicate them to the broader community.

**How would you explain the main finding of your paper?**

The genomes of living organisms are like history books that record their evolutionary past. Sometimes genes can move between very different organisms, for example from bacteria to more complex life forms such as animals, plants or fungi. Previous studies suggested that this might have happened for more than a thousand genes. In our study, we revisited these candidates using larger genomic databases and updated analytical approaches. We found that only about 30% of these genes clearly support this type of transfer. In many other cases, once more species were included in the analysis, the genes turned out to be present in a wider range of organisms than previously known. This shows that our conclusions about gene transfer can change as more genomic data become available, highlighting the importance of continuously re-evaluating evolutionary hypotheses.

… conclusions about gene transfer between very different organisms can depend strongly on the amount of genomic data available and the methods used to analyze them

**What are the potential implications of this finding for your field of research?**

Our findings highlight that conclusions about gene transfer between very different organisms can depend strongly on the amount of genomic data available and the methods used to analyze them. As more genomes from diverse species are sequenced, genes that once appeared to come from bacteria may instead turn out to be more broadly distributed across different groups of organisms. This suggests that some previously proposed transfer events may need to be reconsidered. More broadly, our work emphasizes the importance of revisiting evolutionary hypotheses as genomic databases expand and analytical approaches continue to improve.

**Figure:**
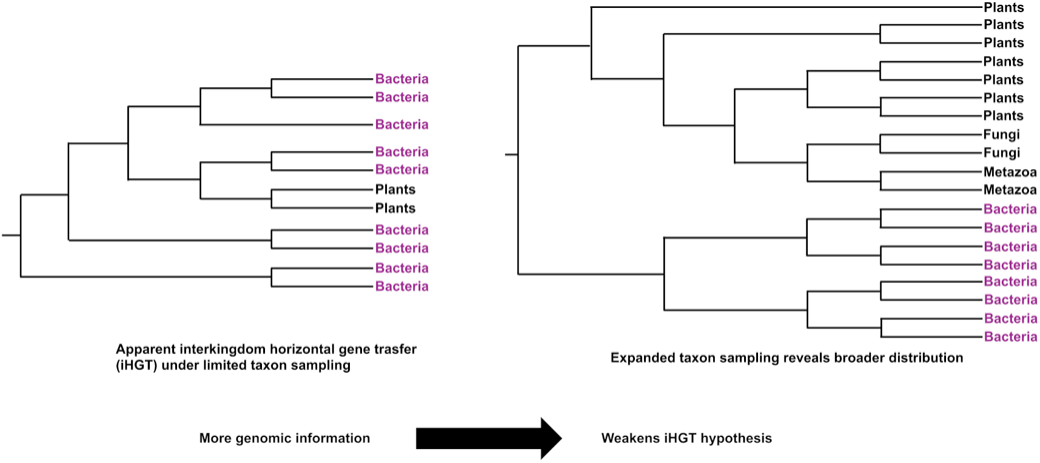
**Conceptual example illustrating how increasing genomic sampling can change interpretations of interkingdom horizontal gene transfer.** With limited data (left), a gene may appear to have been transferred from bacteria to a specific eukaryotic lineage. However, when additional genomes are included (right), homologs from other eukaryotes may be detected, revealing a broader distribution and weakening the original transfer hypothesis.

**Which part of this research project was the most rewarding?**

One of the most rewarding aspects of this project was engaging with a topic that remains actively debated in evolutionary biology. Working on a question where different interpretations exist made the research particularly stimulating. It allowed me to experience an important aspect of science: proposing an interpretation, presenting the evidence, and receiving thoughtful and critical feedback from reviewers and the scientific community. That process of discussion and refinement is essential for advancing our understanding.

**What do you enjoy most about being an early-career researcher?**

What I enjoy most about being an early-career researcher is the opportunity to constantly learn and explore new ideas. At this stage, there is a lot of freedom to develop new skills, collaborate with researchers from different fields and approach problems from fresh perspectives. I find it especially exciting to work at the intersection of biology and computational methods, where new tools and data are continuously opening possibilities for discovery.

**What piece of advice would you give to the next generation of researchers?**

Stay curious, remain humble and find mentors who can guide you. At the same time, learn to be self-directed, because research often requires exploring new ideas independently. I would also encourage young scientists to explore different fields until they discover what truly excites them. Exploration is like opening a gift: you never know exactly what you will find, but the experience of discovery is always rewarding.

Exploration is like opening a gift: you never know exactly what you will find, but the experience of discovery is always rewarding

**What's next for you?**

In the near future, my main goal is to complete my PhD, where I will continue studying horizontal gene transfer and exploring how computational approaches can improve its detection. I am particularly interested in integrating machine learning and deep learning methods to analyze these events more efficiently as genomic data continues to grow. I am also interested in writing about the experiences of early-career researchers in middle- and low-income countries, as navigating scientific careers in these contexts presents unique challenges. In the longer term, I would like to apply my bioinformatics skills to biomedical research, particularly in the study of lymphoma. This interest is personal to me, as my grandmother passed away from this disease, and I would like to contribute in some way to improving our understanding of it. I am always open to collaborations that combine computational approaches with questions in evolutionary biology or biomedical research.
